# Texas State Medical Association

**Published:** 1897-05

**Authors:** 


### EDITORIAL DEPARTMENT.

F. E. DANIEL, M. D., Editor.
S. E. HUDSON, M. D., Managing Editor.
A. J. SMITH. M. D., Galveston, Associate Editor.

EDITORIAL STAFF:

PROF. J. E. THOMPSON, M. D., Texas Medical College, Galveston; Surgery.
PROF. WM. KEILLER, M. D., Texas Medical College, Galveston; Obstetrics and Gynecology.
PROF. DAVID CERNA, M. D., Texas Medical College, Galveston; Therapeutics.
PROF. A. J. SMITH, M. D., Texas Medical College, Galveston; Medicine.
DR. R. H. L. BIBB, City of Mexico; Foreign Correspondent.

Published Monthly at Austin, Texas, by Drs. Daniel and Hudson. Subscription price $2.00 a year in advance.

Eastern Agents: The Monihan-Fairchild Special Agency, 24 Park Place, New York City.

Official organ of the West Texas Medical Association, the Houston District Medical Association, the Austin District Medical Society, Brazos Valley Medical Association, the Galveston County Medical Society, and several others.

### TEXAS STATE MEDICAL ASSOCIATION.

THE PARIS MEETING of the Texas State Medical Association was, numerically, a disappointment to those who expected a large attendance. The 28th annual session was held in that city on 27, 28, 29 and 30 April, ult. There was not a quorum the first morning; but the Secretary and some twenty or thirty delegates arrived on the noon train, and the meeting practically begun with the afternoon session. Later arrivals swelled the number to, perhaps, one hundred. What was lacking in numbers seemed to have been made up in enthusiasm. It was decidedly an interesting meeting, scientifically considered, some noteworthy papers having been read, and the discussions—a transcript of which is promised us for our June number—were interesting and important. I say that those who expected a full meeting were disappointed. The selection of so small a place so remote from the center of the State, and so inaccessible, (it took us twenty-four hours each way—going and coming, Austin to Paris)—we regarded as unfortunate. The reasons assigned for it, however, we admit, were plausible; but the sequel shows that they were not well grounded. It was to give the profession of that section an opportunity of joining. There were, we learn, only about twelve additions to the membership, and all of them not from the immediate vicinity. In fact, North Texas was not as well represented as other portions of the State.

The profession of Paris, we are pleased to say, took the initiative in the matter of omitting all social features of the meeting. A reception the third night, after the President's address, being the only social function authorized, and we are pleased to reflect that in all probability the visit of the Association entailed little or no expense on the citizens,—a precedent we hope to see followed. True, the committee of arrangements exerted themselves to see that every facility for the meeting and for the accommodation of delegates was provided. There was ample hotel accommodation, and the rates were low, (as they should have been, the Lord knows). Our stay of two days and a half at the Lamar house will live in our memory as a night mare. The fare,—the accommodation, was generally pronounced "rocky." And,—how provoking! Just to recall that, at the depot, some half mile distant from the business center, there is a railroad hotel where the *cuisine* and the service are above criticism; the dinner we had there on the eve of departure was,—especially by contrast with the fare at the Lamar, a dream! Delicious! Brother doctors—if you *have* to go to Paris, profit by our misfortune—our ignorance—and go the half mile three times a day for your meals; it will pay you, and you will escape dyspepsia.

But, we have digressed. The usual routine of section work was gone through with with varying interest. The dry details of the minutes are not essential; and we will, later, publish some of the papers and discussions. It is more important now to tell our readers—those who did not attend, what was done at the executive sessions.

The Secretary' report shows the association is not in a flourishing condition financially; it being considerably in debt, the transactions of last year—produced at less cost than any preceeding issue, and paper bound, are not paid for; a voluntary contribution was called for, and $145 was raised. It was resolved that members who do not pay this year's dues by September 1, be dropped from the roll,—(in addition to the number dropped every year for three years' delinquency under the law.) The money is needed to pay for the transactions of this meeting. Dropping members for non-payment of three year's dues has kept the membership down to somewhere between four hundred and five hundred for the last ten or twenty years; and we are fearful that if this summary measure be executed the membership will be still further depleted. But desperate diseases require desperate remedies; and something *had* to be done. The committee passed a vote of thanks to the Secretary for his zeal, and faithful service, and especially for his skillful management of the Association's financial affairs. By those disposed to levity, this might be mistaken for irony; and a member,—miserable pessimist! asked me if they were not guying the Secretary!

The treasurer, Dr. Larendon, having served twenty-five years, nineteen years without compensation—four at $150 a year and the last two at a cut salary of $75, thought the occasion a fitting one to resign, he being, as he expressed it, in the zenith of his fame. The resignation was accepted, but at the earnest solicitation of members not to desert the Association in its extremity, it was reconsidered, and he consented to stand by the mast till the clouds roll by.

The committee having charge of the medical bill reported progress, or, rather, lack of progress of the bill, and an auxiliary committee was appointed to aid in pushing the bill through the legislature. We did not get the names of the committee.

In anticipation of the passage of the bill, the Association, in pursuance of one of its provisions, selected the gentlemen whose names will be submitted to the governor for appointment on the board of medical examiners when the bill becomes a law. (To publish the names at this time would be rather indelicate, perhaps,—in case the bill should finally fail.)

The place of next meeting selected, is Houston. That is sensible, and doubtless there will be a rousing meeting; time, 4th Tuesday in April, 1898. The officers elected are,

President, Professor Bacon Saunders, M. D., Fort Worth.
1st Vice-President, Dr. S. C. Red, Houston.
2nd Vice-President, Dr. A. C. Scott, Temple.
3rd Vice-President, Dr. C. M. Alexander, Coleman.

To serve on the Judicial Council for three years:

Dr. R. R. Walker, Paris.
Dr. S. F. King, Sherman.
Dr. C. S. Bobo, Boyd.
Dr. Taylor Hudson, Belton.

Chairman of Committee of Arrangements, Dr. J. A. Mullen, Houston.

The officers of sections have not yet been announced.

Inasmuch as all the officers for this year are prominent railroad surgeons, the JOURNAL suggests that a *Section on Railroad Surgery* should be added by all means; indeed, it is an oversight that such section was not organized at this meeting.

The Association was honored by a visit from quite a number of distinguished physicians from St. Louis and Denver. Nearly the entire Faculty of Gross Medical College were present, one of them, Dr. LeMond, being a native Texan, and having been made an honorary member at Fort Worth, did the Association the honor to invite and bring with him several of his colleagues, who were given a warm welcome. I do not remember, exactly, whether or not they were all made honorary members, but I was informed that their names had been sent to the Judicial Council for that purpose. These gentlemen contributed largely to the interest and importance of the meeting. Most of them read valuable papers, and all of them took an active part in discussions. Those from Denver, were,—Professor Blair, Professor LeMond, Professor Ball, Professor Hurshley, Professor Beggs, Professor Davis and Professor Macphatter. From St. Louis we had Ex-Prof. E. Lanphear, the celebrated gynecologist and medical editor, Professor Hurlbert, of the defunct Woman's Medical College, and Dr. J. M. Ball, editor of the *Tri-State Medical Journal;* all courteous, genial, jolly companions, whom we were pleased to meet. One suspicious Middle-Texas delegate said: "Look ye here, Daniel,—aint there no danger of these college fellers coming down here into Texas, drummin' off none of our Texas students?" We answered yes, we thought there was; hoped so, any way; [?] but I said, "my friend, you are away off; they are not thinking of students; they are on a lark; wouldn't *look* at a student!"

It is remarkable how a man loses interest in the Association after he has been president! By making our presidents, we drive our best men out of the society; ex-presidents seldom attend. Good old Dr. Sears was the only ex-president in attendance, except Prof. Paine.

Only two representatives from Austin were there, the two editors and publishers of the *Red Back*—Daniel and Hudson—(who represent Before Taking and After Taking, they say). Only three from Galveston—Professor Paine, Mayor Fly, and Secretary—Professor West; not one from San Antonio; not one from Denison, within a stone's throw; one from Clarksville, next door; none from Texarkana; three from Sherman; one from

Bonham; one from Greenville; one single representative from Fort Worth; one from Marshall; six from Dallas!—Oh my, "what a falling off was there, my countrymen"—doctors!

It is to be hoped that the failure of this experiment will be salutary, and that the Association will not again meet on the extreme periphery of the medical circle. It would be much better to meet every year at some central point, like Austin or Waco. Told you so.

The following is a list of new members reported by the Judicial Council: E. D. Peck, Oglesby; W. N. John, Nelson, I. T.; T. M. Wilson, Thornton; T. B. Davis, Grapevine; Sam'l E. Milliken, Dallas; W. D. Cross, Sylvan; J. M. Neel, Bonham; J. M. Woodson, Temple; A. J. Marbury, Ballinger; J. Wes. Carson, Indian Gap; C. E. Cantrell, Wolfe City; J. A. Odom, Quinlan.

